# β-Defensins Coordinate In Vivo to Inhibit Bacterial Infections of the Trachea

**DOI:** 10.3390/vaccines6030057

**Published:** 2018-08-28

**Authors:** Lisa Kathleen Ryan, Jichuan Wu, Kyell Schwartz, Sunghan Yim, Gill Diamond

**Affiliations:** 1 The Public Health Research Institute Center, Rutgers New Jersey Medical School, Newark, NJ 07103, USA; 2 Division of Pulmonary and Critical Care Medicine, Department of Medicine, Rutgers New Jersey Medical School, Newark, NJ 07103, USA; 3 Department of Oral Biology, Rutgers New Jersey Dental School, Newark, NJ 07103, USA; jichuan.wu@temple.edu (J.W.); kyellschwartz426@gmail.com (K.S.); yimsu75@gmail.com (S.Y.); gdiamond@dental.ufl.edu (G.D.)

**Keywords:** β-defensins, *Bordetella bronchiseptica*, epithelial cells, innate immunity

## Abstract

β-defensins are predicted to play an important role in innate immunity against bacterial infections in the airway. We previously observed that a type III-secretion product of *Bordetella bronchiseptica* inhibits the NF-κB-mediated induction of a β-defensin in airway epithelial cells in vitro. To confirm this in vivo and to examine the relative roles of other β-defensins in the airway, we infected wild-type C57BL/6 mice and mice with a deletion of the mBD-1 gene with *B. bronchiseptica* wild-type strain, RB50 and its mutant strain lacking the type III-secretion system, WD3. The bacteria were quantified in the trachea and the nasal tissue and mRNA levels of mouse β-defensin-3 (mBD-3) were assessed after 24 h. Infection with the wild-type bacterial strain resulted in lower mBD-3 mRNA levels in the trachea than in mice infected with the type III-deficient strain. Furthermore, we observed an increase in bacterial numbers of RB50 only in the tracheas of mBD-1-deficient mice. Neutrophils were also more abundant on the trachea in RB50 infected WT mice but not in the bronchiolar lavage fluid (BAL), compared with WD3 infected WT and mBD-1^−/−^ mice, indicating that the coordination of β-defensin chemotactic effects may be confined to tracheal epithelial cells (TEC). RB50 decreased the ability of mice to mount an early specific antibody response, seven days after infection in both WT and mBD-1^−/−^ mice but there were no differences in titers between RB50-infected WT and mBD-1^−/−^ mice or between WD3-infected WT and mBD-1^−/−^ mice, indicating mBD-1 was not involved in induction of the humoral immune response to the *B. bronchiseptica*. Challenge of primary mouse TEC in vitro with RB50 and WD3, along with IL-1β, further corroborated the in vivo studies. The results demonstrate that at least two β-defensins can coordinate early in an infection to limit the growth of bacteria in the trachea.

## 1. Introduction

Bacteria that cause infections in the airway must evade a number of host defense mechanisms that normally combine to prevent these microorganisms from colonization (reviewed in [1,2,3]). One component of this defense is the production of antimicrobial peptides such as β-defensins in the tracheal mucosa, which are either constitutively produced, or induced by cytokines and other bacterial components such as lipopolysaccharide (LPS) via regulation by the transcription factor, NF-κB [4,5,6,7,8].

Previously, several studies have demonstrated that introducing bacterial pathogens can induce gene expression of β-defensins in airway epithelial cells (AEC) both in vitro and in vivo, through activation of innate immune pathways leading to the activation of NF-κB-mediated gene expression [9,10,11,12,13,14,15,16]. Several studies have also revealed that β-defensins are induced in the mucosa during chronic states of disease induced by bacteria [17,18,19,20]. 

To support the hypothesis that β-defensins are important in airway defense in vivo, two studies showed some delayed clearance of *Haemophilus influenzae* [21] and no significant effect on infection of *Staphylococcus aureus* and *Streptococcus pneumoniae* [22] in mice lacking mouse β-defensin-1 (mBD-1). Because of the minor effect of deleting one β-defensin gene on bacterial infection, it was thus suggested that multiple β-defensins may cooperate to provide innate immune host defense [19]. 

Demonstrating this cooperation in vivo has been difficult experimentally in mice, due to the close genetic linkage of the β-defensins [23], which does not lend to the development of mice deficient for more than one gene. In order to address this, we made use of the ability of the airway pathogen, *Bordetella bronchiseptica*, RB50, which colonizes airway epithelial cells and expresses a type III secretion factor that inhibits NF-κB activation in the host cell and a *B. bronchiseptica* mutant strain lacking the type III secretion system and factor, WD3 [24,25,26]. 

Our research has demonstrated that the active type III secretion system was sufficient to inhibit the bacteria-mediated induction of the bovine homologue to human β-defensin-2, tracheal antimicrobial peptide (TAP), in cultured bovine tracheal epithelial cells (TEC), whereas the WD3 mutant did not inhibit the induction of TAP [12]. This occurred due to the inhibition of NF-κB activation, which TAP gene expression depends on [5]. In this study, instead of TAP, we targeted the hBD-2 and TAP homologue, mouse β-defensin-3 (mBD-3), which is governed by the same transcription factor (NF-κB). Thus, the suppression by RB50 is not at the gene level but in signal transduction of mBD-3 production by NF-κB. Since the type III secretion factor can inhibit NF-κB, the end result should be a suppression of mBD-3 transcription, based on the regulation of TAP by NF-κB [5,12]. We also took advantage of the deletion of the mBD-1 gene in mice. mBD-1 is not regulated by NF-κB, so it is not affected by RB50 [27]. To examine the potential for multiple β-defensins to contribute to an initial defense of the airway, we infected mice deficient in mBD-1, the murine homologue to human β-defensin-1 (hBD-1) [21,28,29] with the wild type (WT) *B. bronchiseptica* strain, RB50, thus allowing the suppression of two β-defensins simultaneously. Because TEC express the greatest levels of inducible β-defensins [30] and *B. bronchiseptica* infects and colonizes on TEC [12], our study focused on the trachea rather than the whole lung. Our results demonstrate that in the trachea, multiple β-defensins coordinate together to contribute to enhance airway host defense against bacteria.

## 2. Materials and Methods

### 2.1. Bacterial Strains and Growth

Wild-type (RB50) and mutant (WD3) strains of *B. bronchiseptica* [12,24,31] were cultured on Bordet-Gengou agar (Difco, Detroit, MI, USA) supplemented with 5% sheep blood (Quad Five, Ryegate, MT, USA) and 1% glycerol and 20 µg/mL Streptomycin, as described [12]. Liquid cultures were grown in modified Stainer-Scholte medium [24]. In order to activate BvgAS and to maintain the bacterial strains in Bvg^+^ phase, the bacterial colonies and liquid cultures were grown to mid-log phase at 37 °C and liquid cultures were maintained at 37 °C prior to cell culture infection.

### 2.2. Animals

C57Bl/6 female mice, 12–16 weeks old, were purchased from Taconic Laboratories, (Albany, NY, USA) as 6–8 week old mice and housed in an SPF barrier facility at Rutgers New Jersey Medical School. Mice were utilized and cared for according to an approved by Institutional Animal Care and Use Committee (IACUC) (Number: 05040E0708). Sterilized water and rodent chow were given ad libitum. mBD-1^−/−^ mouse breeding pairs were obtained from James M. Wilson under an MTA from the University of Pennsylvania and bred at the transgenic breeding facility at Rutgers New Jersey Medical School. Tail clippings were analyzed by PCR for mBD-1 to ensure the correct genotype for WT and mBD-1 deletion [21].

### 2.3. Infection of Mice

**Figure 1:**
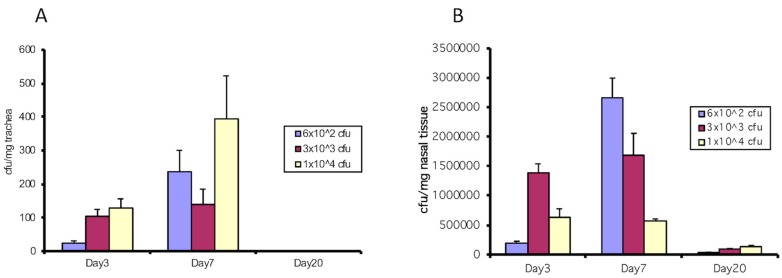
Colonization with *Bordetella bronchiseptica* wild type strain (RB50) in the trachea and nose in C57Bl/6 wild type mice (WT) during the course of infection. Mice were intranasally inoculated with varying cfu (600, 3000 and 10,000) in 50 µL PBS and tracheas and nasal epithelium were excised after 3, 7 and 20 days. Tissues were homogenized, cultured and cfu/mg trachea (**A**) and cfu/mg nasal tissue (**B**) were determined. Increasing inoculums led to increased cfu with RB50. Colonization was evident at 3 days, peaked at 7 days and was nearly gone by 20 days with these doses.

**Figure 2:**
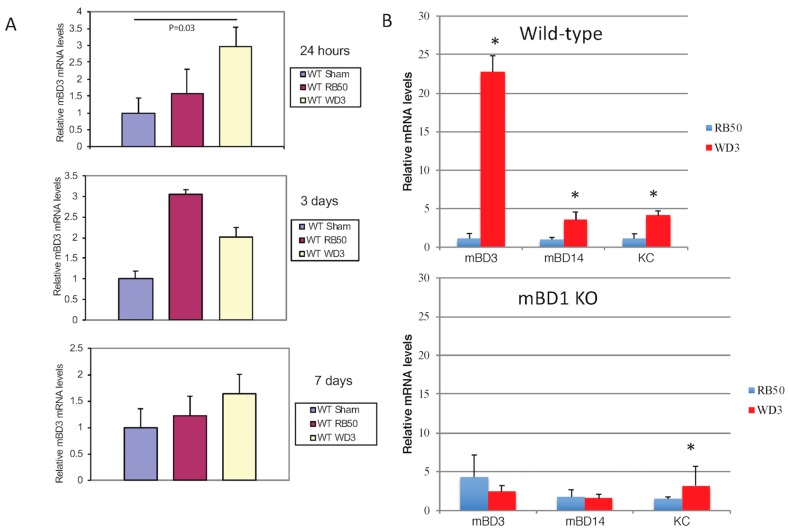
In vivo inhibition of β-defensin expression by the type III secretion system of *B. bronchiseptica*. (**A**) Wild-type mice (C57Bl/6) were intranasally inoculated with 1 × 10^4^ cfu wild-type bacteria (RB50) or its type III secretion deficient (WD3) strain in 50 µL (or 50 µL PBS). After 24 h, 3 days and 7 days, tracheas were excised, mRNA was extracted and relative mBD-3 levels were quantified by qRT-PCR, normalized to β-actin. Line or asterisk indicates a significant increase in mBD-3 mRNA after 24 h and at 3 days (*p* ≤ 0.05). (**B**) Wild-type mice (C57Bl/6) were intranasally inoculated with 1 × 10^5^ cfu wild-type bacteria (RB50) or its type III secretion deficient (WD3) strain in 50 µL (or 50 µL PBS). After 24 h, tracheas were excised, mRNA was extracted and relative mBD-3, mBD-14 and KC levels were quantified by qRT-PCR. Asterisk denotes a significant difference between RB50 and WD3 infections as determined by Student’s *t*-test (*p* ≤ 0.05). Error bars = SEM.

Groups of five mice were anaesthetized by injection I.P. with 50 mg/kg ketamine/20 mg/kg xylazine in 0.1 mL. A suspension of 2 × 10^6^ bacteria/mL in sterile PBS, or PBS alone as a sham infection, were introduced into the mice in a 50 µL total volume intranasally with a 200 µL Eppendorf pipette, pipetting about 5 µL per breath per nostril (25 µL per nostril total volume). The inoculum was cultured as described above to ascertain the number of bacteria inoculated intranasally into each mouse. The total number of bacteria per mouse = 1 × 10^5^ cfu for most inoculations. Initially, mice were infected with lower numbers of bacteria (cfu) as indicated in Figure 1 (600, 3000 and 1 × 10^4^) (see Section 3) and Figure 2A (1 × 10^4^) (see Section 3). Mice were sacrificed using sodium Nembutal. Tracheas were excised, measured and divided in half. One half of the trachea was homogenized for mRNA isolation and assessment (see Figure 2A in Section 3). The other half of the trachea was homogenized in sterile PBS, diluted serially in 10-fold dilutions, spread with a sterile glass spreader and plated onto BG agar containing 5% sheep blood, 1% glycerol and 20 µg/mL streptomycin. Colonies (small, opaque and slightly hemolytic) were observed and counted after incubation at 37 °C for 2–3 days. Blood was collected upon exsanguination for serum analysis of antibodies to *B. bronchiseptica*. In other infections only lasting 24 h, mice were infected with 1 × 10^5^/mouse of either RB50 or WD3 (to ensure colonization and production of the type III secretion factor within this short time) and 24 h later, whole tracheas were excised and processed for mRNA analysis (see Figure 2B in Section 3) or bronchoalveolar lavage (see Figure 5 Section 3) and in another experiment, tracheas were excised on Days 1, 3 and 7 for histopathology (see Figure 4 in Section 3). 

### 2.4. Histopathology

Lungs and trachea were excised from infected mice were fixed in 4% paraformaldehyde and processed for histology. Slides were stained with H&E and observed by light microscopy for gross tissue damage and neutrophil influx. Neutrophil (PMN)s were identified histologically by banded or segmented dark purple nuclei, round shape, size and the lack of proximity and organization to other cells (single cells).

### 2.5. In Vitro Infection of Differentiated Tracheal Epithelial Cells

Tracheas were excised from either C57BL/6 wild-type (WT) or mouse β-defensin-1 (mBD-1)^(−/−)^ (KO) mice using sterile surgical techniques. Tracheas were soaked and washed in ice-cold F-12 medium with 1% penicillin/streptomycin (Invitrogen). The tissue was digested using 1.5 mg/mL pronase in the F-12 medium for 18 h at 4 °C. Fetal bovine serum (FBS) was then added to the medium to give 10% final concentration. Tracheas were washed 3× in 3 tubes containing 3 mL of this medium. Tracheas were discarded and the contents of the tubes were pooled and centrifuged at 400× *g* for 10 min at 4 °C. Cells were re-suspended in 200 µL/trachea of F-12/pen-strep/0.5 mg/mL crude pancreatic DNase I and 10 mg/mL BSA, incubated on ice for 5 min and centrifuged at 400× *g* for 5 min at 4 °C. Cells were re-suspended in BEGM medium (Lonza) with 10% FBS, plated on a tissue culture plate for 3–4 h and incubated at 37 °C with 5% CO_2_. The adherent cells were discarded and the non-adherent cells were collected and centrifuged as above. Cells were counted with 0.4% trypan blue and a hemocytometer and re-suspended in BEGM medium and seeded in a collagen-coated 12 well transwell plate at 8.4 × 10^4^ cells/well. When cells were confluent, the medium on the apical side of the transwell was removed (about 1 week later) to allow differentiation of the cells above the airway liquid interface (another week). Collagen-coated 12 well plates were made by dissolving 500 µg/mL rat tail collagen in 0.1% acetic acid, then diluting the stock 1:10 in water, filter sterilizing and coating 200 µL/filter. Filters are dried overnight in the hood (open top), then sterilized for 30 min in UV light prior to cell seeding. Media was changed after 24 h and every other day until cells were confluent. (protocol modified from [32]. Differentiated cells were challenged with either 100 ng/mL IL-1β in the basolateral medium or with live *B. bronchiseptica* (strains RB50 and WD3, MOI = 1000:1) on the apical surface for 6 h. Total mRNA was isolated and levels of mBD-3 and mBD-14 mRNA were quantified by qRT-PCR. *n* = 3 cultures; error bars = ±SEM. Statistical comparison was done using the Student’s *t*-test with significance at *p* ≤ 0.05.

### 2.6. Quantification of mRNA Levels

Tissues were homogenized as per manufacturer’s instructions with mini-homogenizers in RLT buffer (Qiagen, Valencia, CA, USA). Total RNA was isolated using the RNeasy Mini Kit with on-column DNase (Qiagen) and stored at −80 °C until analysis. 500 ng of total RNA was reverse transcribed with Superscript III reverse transcriptase by oligo(dT) primers in 20 µL as described by manufacturer protocol (Invitrogen, Carlsbad, CA, USA). No RT and no RNA controls were also analyzed. For the qPCR reaction, 500 ng of cDNA was transcribed with 1× SYBR Green PCR Master Mix and 300 nM primers for mBD-3, mBD-4, mBD-14, KC, β-actin, or β2-µglobulin primers (300 nM forward, 50 nM reverse), in a final volume of 20 µL (For primer sequences, see Table 1). Annealing temperatures for all primers was 60 °C on a Bio-Rad my iCycler thermocycler.

### 2.7. ELISA for Specific Antibody against B. bronchiseptica

Blood samples were obtained upon sacrificing the mice on days 3, 7 and 20. Samples were allowed to clot and were centrifuged at 300× *g*. ELISA was performed utilizing a modification of the method of [33]. Serum samples from either wild-type (WT), heterozygous (HT) or mBD-1 knockout (KO) mice (*n* = 5 per group) were serially diluted, starting at 1:100 across the plates after coating ELISA plates overnight at 4 °C with either heat-killed RB50 or WD3 and incubating diluted serum samples in PBS-Tween 20 for 2 h at 37 °C. Following serum incubation, plates were washed with PBS-Tween 20 three times, then incubated with 1:1000 HRP-conjugated goat anti-mouse immunoglobulin M, G and A (combined to capture the entire response) for 1 h at 37 °C and washed three times again. Enzyme detection used ABTS (Sigma, St. Louis, MO, USA) substrate for 30 min and O.D. at 450 nm was measured. Specific antibody titer was determined by averaging duplicates of O.D. readings from each serum sample, then comparing the average O.D. of each dilution to the baseline O.D. of the normal mouse serum control, diluted 1:100 (0.051). The titer was the highest dilution of antibody giving an O.D. reading of double the baseline O.D. (normal serum). The titers of 5 mice in each group were averaged and the variation was reported as the Standard Error of the Mean (SEM) antibody titer. 

### 2.8. Bronchoalveolar Lavage (BAL) Cell Morphology

Mice were infected with 1 × 10^5^ RB50 or WD3 intranasally as described above and after 24 h, lavaged with a 1 cc syringe with Hank’s Balanced Salt Solution without Ca^2+^ and Mg^2+^ (HBSS^−−^, InVitrogen, Carlsbad, CA, USA). BAL cells were pelleted at 300× *g*, diluted 1:10 in HBSS^−−^, cytospun onto a slide and stained with the Diff-Quik stain kit (VWR, Radnor, PA, USA). One hundred cells were differentially counted for macrophages, neutrophils (PMN) and lymphocytes. Lymphocytes were small and have a large dark blue single nucleus and very little cytoplasm (light blue). Monocytes were larger, have a single dark blue nucleus and have a larger amount of cytoplasm, stained light blue. PMN were identified by their banded or segmented dark blue nucleus and lavender cytoplasm with violet granules. Eosinophils are identified by their segmented nuclei and orange/pink granules in the cytoplasm but none were observed. Viable cells were also counted on a hemocytometer following staining with 0.4% trypan blue dye (ThermoFisher, Life Technologies, Grand Island, NY, USA). Cells that excluded the dye were deemed viable. Total number and percentage of each cell type was averaged and standard error of *n* = 5 mice was calculated.

### 2.9. Statistics

Bacterial numbers (cfu/mg tissue), mRNA levels, immunoglobulin levels and BAL cell morphology counts were compared by calculating mean + standard deviation (*n* = 5) within the experiment and comparing them by Student’s *t*-test, with significance set at *p* ≤ 0.05. 

## 3. Results

### 3.1. The Type III Secretion System of B. bronchiseptica Inhibits mBD-3 Expression in Mouse Trachea

To support our in vitro results [12] with in vivo experimentation, we intranasally infected wild-type C57/BL6 mice with two strains of *B. bronchiseptica*, RB50 and the type III-deficient strain, WD3. We first determined the effective inoculation that established infection. Figure 1 demonstrates various doses of 6 × 10^2^, 3 × 10^3^ and 1 × 10^4^/mouse of *B. bronchiseptica* RB50 in C57Bl/6 mice (WT) in the trachea and nose. Infection was best utilizing 1 × 10^4^/mouse and cfu numbers were evident by Day 3 and peaked on Day 7 following infection. After 20 days, cfu counts were only in the nasal tissue and not in the trachea. 

Due to the early action of β-defensins on an initial infection, we also had a group of WT mice that was sacrificed after 24 h after infection with either PBS (sham control), RB50, or WD3 strains. After 24 h of infection with 1 × 10^4^, mice were sacrificed and tracheas were excised. The tracheas were split in half, to allow for isolation of mRNA and bacteria. Figure 2A shows that mBD-3 mRNA in WT mice infected with 1 × 10^4^ WD3 was significantly elevated compared with sham-infected controls and there was a trend toward impeded mBD-3 upregulation of mRNA in mice infected with 1 × 10^4^ RB50. After 3 days, mBD-3 went up significantly with both RB50 and WD3 compared with the sham controls but RB50-induced mBD-3 was significantly elevated compared with WD3. By Day 7, this elevated mBD-3 mRNA by both strains disappeared in the 7 day group samples and mBD-3 gene expression was not significantly different from controls. Bacterial colonies began to colonize in the tracheas of WT mice at 24 h in this experiment—a few hundred/mg trachea for RB50 and approximately 1500/mg trachea for WD3 (data not shown).

To determine the relative contribution of two β-defensins, we obtained a strain of mouse deficient in the constitutive β-defensin-1 (mBD-1, which is the homolog of hBD-1) [21], mBD-1^(−/−)^. Figure 2B demonstrates one separate infection experiment with a higher inoculum, 1 × 10^5^/mouse RB50 or WD3, in both WT and mBD-1^(−/−)^ mice. A 10-fold higher inoculum than the previous experiment was used to ensure the effect of the type III secretion factor at 24 h. For this experiment, whole tracheas were utilized for total RNA extraction. When β-defensin mRNA was quantified with RT-PCR, we observed a statistically significant difference (Student’s *t*-test, *p* < 0.05) between the relative mBD-3 mRNA levels in tracheas from mice infected with the wild-type RB50 strain compared with the type III secretion-deficient WD3 strain (Figure 2B). We repeated the infection twice in mice heterozygous (HT) for mBD-1 (F1 cross between C57Bl/6 wild type and mBD-1^(−/−)^) and found similar results: WD3 had a significant induction of mBD-3 mRNA levels from tracheal cells excised 24 h after infection, whereas infection with RB50 containing the type III secretion system generated mBD-3 mRNA levels similar to a sham infection with PBS (data from sham and the two other experiments with HT mice not shown). In the mBD1^(−/−)^ mouse, mBD-3 mRNA levels were elevated but not to the levels that WD3 induced in WT mice (3-fold in mBD1^(−/−)^ versus 23-fold in WT mice) and the lower levels of mBD-3 mRNA remained with the RB50 strain infecting the mBD1^(−/−)^ mice. There was an increase in mBD-14 and keratinocyte cytokine (KC), a homologue of IL-8 in mice, in both WT and mutant mice. Like mBD-3 mRNA, RB50 significantly suppressed the induction of mBD-14 and KC in WT mice. In mBD1^(−/−)^ mice, only RB50-induced KC was significantly lower than WD3-induced KC, whereas there were no differences between RB50 and WD3 in their induction of β-defensins mBD-3 and mBD-14.

### 3.2. A Deficiency in mBD-1 Results in an Increased Colonization by Wild-type B. bronchiseptica

**Figure 3:**
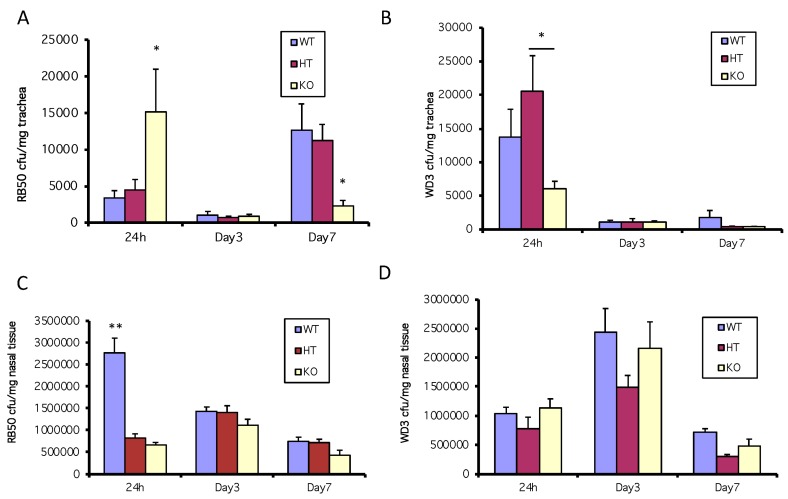
In vivo coordination of β-defensin activity to inhibit bacterial colonization in the trachea but not the nose in the first 24 h. 10^5^ cfu of *B. bronchiseptica* RB50 or WD3 were intranasally inoculated into WT and mBD-1-deficient mice. After 24 h, tracheas and nasal tissue were excised, homogenized and plated on selective media to enumerate *B. bronchiseptica* colonies. (**A**) RB50, trachea; (**B**) WD3, trachea; (**C**) RB50, nose; (**D**) WD3, nose. * denotes significant increase in mBD1^−/−^ tracheas vs. WT and HT and in HT vs. KO (*p ≤* 0.03); ** significant increase in WT nasal tissue vs. HT and KO (*p* < 0.0002). Error bars = SEM.

Since the results shown in Figure 2B suggest that an active type III secretion system inhibits the bacteria-mediated induction of mBD-3, the murine homolog of hBD-2, we infected WT, HT or mBD-1-deficient mice (15 per mouse strain/bacterial strain combination) with the wild type (RB50) or mutant strains (WD3) of *B. bronchiseptica* and sacrificed groups of 5 of each type of mice at each time point, excising the trachea and nasal tissue. In one experiment, using a 1 × 10^5^ inoculum of the wild-type strain of bacteria, RB50, when both the constitutive form of β-defensin (mBD-1) is suppressed (by genetic knockout) and the inducible form is suppressed (by the active type III secretion system of RB50), there is an increase in susceptibility to bacterial colonization in the trachea in mBD-1^(−/−)^ mice (yellow bar) compared with that of WT and HT mice (blue and red bars) infected with RB50 at 24 h (Figure 3A). At 24 h, mice infected with a 1 × 10^5^ inoculum of the mutant *B. bronchiseptica* strain, WD3, had 1.4 × 10^4^ bacteria/mg trachea in the WT mice and 6 × 10^3^ in the mBD-1^(−/−)^ mice but there were no significant differences in colonization between C57Bl/6 WT and the mBD-1^(−/−)^ mice. However, the HT mice had significantly more WD3 cfu/mg trachea compared with the mBD-1^(−/−)^ mice (Figure 3B). In WT mice (blue bars), at 24 h following inoculation with 1 × 10^5^ cfu of RB50 or WD3, WD3 colonized 3-fold more cfu/mg tracheal tissue than in WT mice infected with RB50. In mBD-1^(−/−)^ mice (yellow bars), cfu/mg tracheal tissue was 3-fold more with RB50 compared with mBD-1^(−/−)^ mice infected with WD3.

However, in a separate experiment using a lower inoculum dose of 3 × 10^3^ RB50, the number of bacterial colonies/mg tracheal tissue at 24 h did not vary between strains and diminished to <1000 cfu/mg after 3 days and continued to be <1000 cfu/mg trachea at 7 days after infection (data not shown). 

In the nose (from the first experiment in this section above), bacterial numbers reached almost 3 × 10^6^ cfu/mg nasal tissue after 24 h following the 1 × 10^5^ inoculum of RB50 in C57Bl/6 WT mice, with HT and mBD-1^(−/−)^ mice having colonized a third less RB50, which was significant (Figure 3C). By days 3 and 7, RB50 cfu/mg nasal tissue reached equal levels between the three mouse strains at approximately 1–1.5 × 10^6^ cfu/mg (Figure 3C). In the nose, WD3 cfu/mg nasal tissue was not significantly different between all three types of mice at all time points but the WD3 cfu/mg nasal tissue was approximately a third of the RB50 cfu/mg nasal tissue in WT mice (Figure 3D).

### 3.3. mBD1-Deficient Mice have a Reduced Neutrophil Accumulation in the Trachea in Response to Bacterial Infection

**Figure 4:**
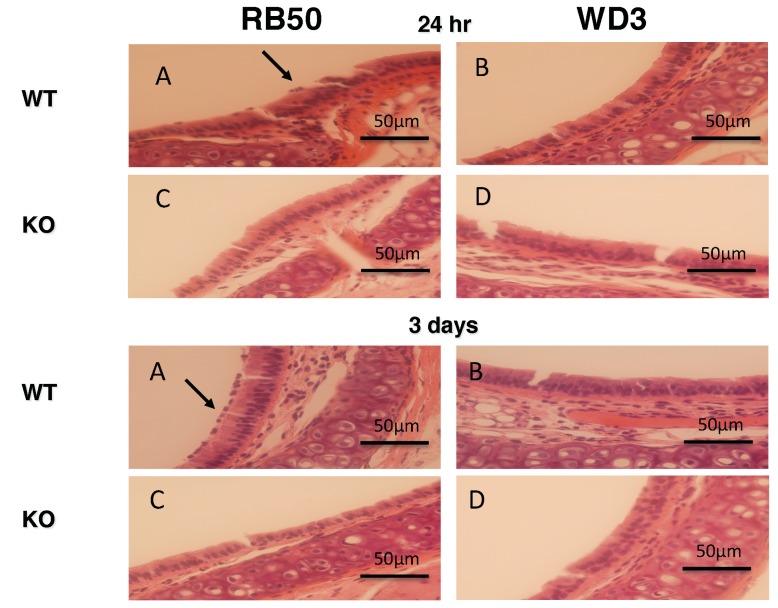
Neutrophil influx in bacteria-infected tracheas in wild-type (WT) and in mBD-1-deficient mice. 10^5^ cfu *B. bronchiseptica* strain RB50 (**A**,**B**) or WD3 (**C**,**D**) were intranasally inoculated into WT (**A**,**C**) or mBD-1-deficient (**B**,**D**) mice. After 24 h, tracheas were excised, fixed and processed for histology. Tissue sections were stained with H&E and examined by light microscopy. Neutrophils were most visible on the surface of tracheas of wild-type mice infected with RB50, with the greatest number occurring at Day 3 after infection. Magnification = 400×.

Since β-defensins are known to exhibit immunomodulatory activities, including chemotactic activity for a variety of host defense cells [34,35,36], we examined the infected tracheal tissue histologically in a separate experiment infecting both WT and mBD-1^(−/−)^ mice with 1 × 10^5^ of each bacterial strain. While WT mice respond to the presence of RB50 with an influx of neutrophils, this influx was very low (2–6/field) in mBD-1-deficient mice (Figure 4), regardless of the type III secretion factor status. Neutrophils were observed on the trachea as early as 24 h (20–45/field) and by three days, as many as 60–105/field on the tracheal apical (airway) surface were observed in WT mice infected with RB50. Few neutrophils were observed on tracheas of either strain of mice infected with WD3, even after 3 days (2–6/field). By Day 7, an average of 20 neutrophils/field were observed with RB50 infected WT mice and 10 neutrophils/field were observed with WD3 infected WT mice. By Day 7 after infection with WD3 in WT and with RB50 in mBD-1^(−/−)^ mice, 1–2 neutrophils/field were observed. Finally, on Day 7, mBD-1^(−/−)^ mice infected with WD3 displayed 20 neutrophils/field.

**Figure 5:**
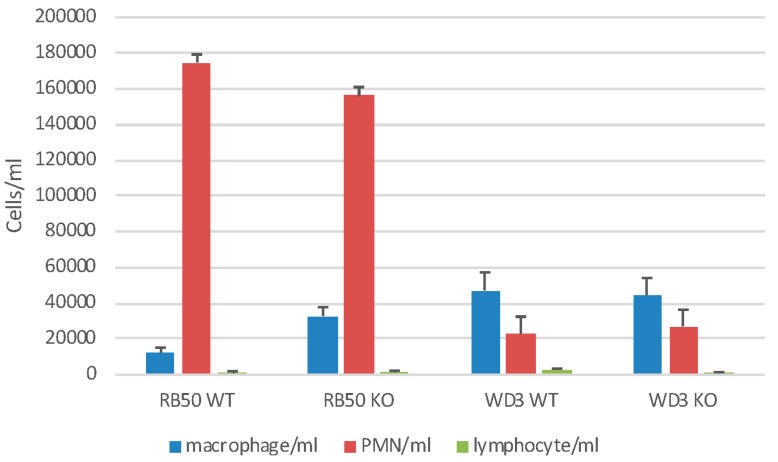
Neutrophil influx into the airways of infected mice. Wild type C57Bl/6 mice or mBD-1^(−/−)^ mice were infected with 1 × 10^5^ RB50 wild type bacteria or WD3 mutant bacteria as indicated. Mice were lavaged with HBSS-24 h following infection. Bronchoalveolar lavage (BAL) cells were stained with trypan blue dye and viable cells counted. Viability was >95%. Cell morphology was determined on cytospun BAL cells via Diff-Quik stain. Alveolar macrophages are indicated by blue bars, neutrophils by red bars and lymphocytes by green bars. Sham-infected mice had 1 × 10^5^ total cells/mL BAL, with 98% alveolar macrophages and did not differ between WT and mBD-1^(−/−)^ mice.

We also examined gross cellular morphology in the whole lung following infection in a separate experiment with 1 × 10^5^ of RB50 or WD3. After 24 h, mice were lavaged with 1 mL of HBSS^−−^ (BAL) (Figure 5). There was a neutrophil (PMN) influx of approximately 1.6 × 10^5^/mL BAL but there were no differences in the recruitment of PMN to the lung between WT and mBD-1^(−/−)^ mice infected with RB50. In mBD-1^(−/−)^ mice, PMN influx was significantly diminished to 3 × 10^4^ with WD3 infection. Sham-infected mice showed no PMN influx (data not shown). Alveolar macrophage (1.1–2.59 × 10^4^/mL) and lymphocyte numbers (4.5–9.3 × 10^2^/mL) did not change significantly with any treatment. The viability of BAL cells was >99%. Baseline, sham-infected WT and mBD-1^−/−^ mice had a total cell count of 1 × 10^5^ viable cells of which 98% were alveolar macrophages.

### 3.4. Early Specific Antibody Formation is not Affected by β-defensin-1

**Figure 6:**
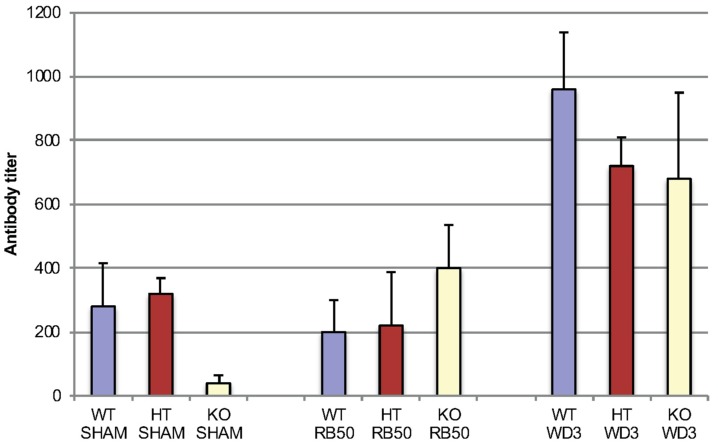
Early serum antibody titers in mice infected with RB50 or WD3 strains of *B. bronchiseptica* seven days after infection (ELISA). Blood was collected from WT, HT and mBD-1^−/−^ mice infected with 1 × 10^4^ RB50 or WD3 for serum IgM, IgG and IgA combined antibody titers against either strain of *B. bronchiseptica* using a secondary antibody that detects all three Ig subtypes. Blood was collected on days 3, 7 and 20 following infection. Black bars indicate the denominator of mean antibody titer in WT mice, blue bars indicate that of HT mice and red bars indicate that of mBD-1^−/−^ mice. Error bars indicate SEM (*n* = 5). Infection was the same infection as Figure 1.

We tested early specific antibody titers (IgM, IgG and IgA together) in mice infected with 1 × 10^4^ RB50 and WD3 in all three strains of mice: WT, HT and mBD-1^(−/−)^, focusing on Day 7 (Figure 6). This was the same infection experiment as Figure 1. No titer was detected on Day 3. After 7 days, WD3 infected mice showed an increase in specific immunoglobulin titer compared with sham-infected mice. WD3-infected mice also showed an increase in titer in WT and HT mice compared with RB50. However, between mouse strains, no differences were observed in titers of mice infected with either RB50 or WD3 at Day 7 after infection. By Day 20, RB50-infected WT mice showed specific antibody titers > 1:2560. We did not test specific immunoglobulin types.

### 3.5. Variable Induction of β-defensins in Tracheal Epithelial Cells In Vitro

**Figure 7:**
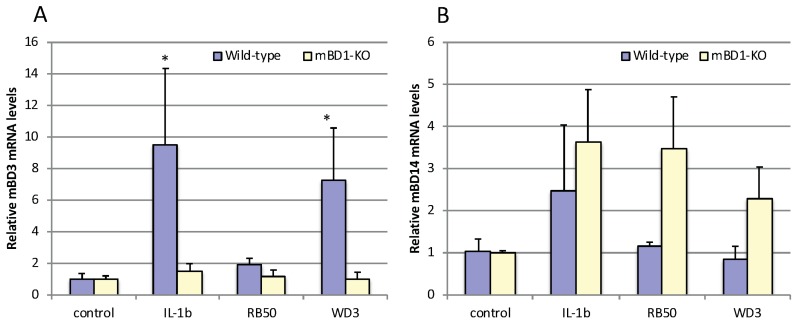
In vitro challenge of primary mouse tracheal epithelial cells from WT and mBD-1^−/−^ mice with RB50, WD3 and IL-1β. Tracheas were excised from either WT or mBD-1^−/−^ mice and grown on airway-liquid interface cultures, allowing differentiation of the tracheal epithelial cells (TEC). 100 ng/mL IL-1β, or 1,000:1 MOI of RB50 or WD3 was added to the apical surface for 6 h. Total mRNA was isolated from the TEC and levels of mBD-3 (**A**) and mBD-14 (**B**) mRNA were quantified by qRT-PCR. *n* = 3 cultures; error bars = +/− SEM. Statistical comparison was done using the Student’s *t*-test with significance (*) at *p ≤* 0.05.

To examine the interaction with the two strains of *B. bronchiseptica* in WT and mBD-1^(−/−)^ mice in the trachea in vitro, tracheas were excised from naïve mice and TEC were cultured for several weeks until they were differentiated. Cells were exposed to IL-1β, RB50 and WD3 for 6 h, then assessed for mBD-3 or mBD-14 mRNA modulation compared with unexposed control cells. Figure 7 shows, as expected, IL-1β stimulated a significant induction of mBD-3 mRNA in WT mice. However, in mBD-1^(−/−)^ mice, mBD-3 mRNA levels were not induced by IL-1β above control, whereas mBD-14 levels were not significantly induced in WT and mBD-1^(−/−)^ mice above control but there was an equal trend towards mBD-14 induction in both types of mice by IL-1β. With RB50 challenge, there was no significant induction of mBD-3 induction in either strain of mice. However, with WD3 in WT mice, as it was with infection with WD3 in vivo, mBD-3 was significantly induced compared with mRNA levels of TEC challenged with RB50. In mBD-1^(−/−)^ mice, WD3 and RB50 did not induce significant levels of mBD-3 mRNA above control. RB50 and WD3 did not induce mBD-14 in WT mice. However, in mBD-1^(−/−)^ mice, as with IL-β, both RB50 and WD3 induced mBD-14 two to four-fold compared with unchallenged TEC, which was not significantly different from control.

## 4. Discussion

β-defensins are widely expressed in epithelia and have been proposed to play an important role in the host defense against microbial pathogens. This was first supported by the observations both in vitro and in vivo that their genes are induced by microbe-associated molecular patterns, such as lipopolysaccharide (LPS) [1]. There are some associations observed with diseases, mostly of single nucleotide polymorphisms and a variety of infectious diseases [37,38,39,40,41], as well as variations in β-defensin copy number [42,43] but there are only a few in vivo experimental studies to support the roles of β-defensins in antibacterial host defense [8,19,20]. Wilson et al. took advantage of the fact that α-defensins are proteolytically processed to the active mature form by a single enzyme, the metalloproteinase, matrilysin. Deletion of this enzyme in mice led to an increased susceptibility to colonization of the gut [44]. In contrast, β-defensins do not appear to be subjected to secondary processing after signal peptidase [45] and thus this method would not be applicable.

Until recently, the sole β-defensin knockout demonstrated to date had been that for mBD-1 [21,22]. This deletion is specific for only mBD-1 and thus, mBD-3 is unaffected, despite its close proximity on chromosome 8 [46]. Initial experiments on those mice suggested that although mBD-1 may play a role in the initial stages of bacterial infection [21,22], the other β-defensins expressed in the airway may also contribute to the overall innate immune defense [9,10,11,12,13,14,15]. Recently, a mouse with an mBD-3 deletion has been created to study the protective role of mBD-3, mBD-4 and cathelin-related antimicrobial peptide (CRAMP) in a model of *Fusarium solani* keratitis [47]. In this model, disease severity was significantly enhanced and progressed quickly in concert with increased neutrophil recruitment (important in fungal infections) and delayed elimination of the fungi compared to controls. Studies are yet to be done with bacteria infecting mBD-3^−/−^ mice. 

Although mBD-1 has little effect with bacterial infections, this β-defensin may be more important in the pathogenesis of viral infections, for infecting mBD-1^−/−^ mice with mouse-adapted Hong Kong 1968 influenza A (H3N2) led to greater lung inflammation, body weight loss and mortality compared with WT C57Bl/6 mice [48]. The regulation of hBD-1 appears to be via an interferon regulatory pathway (IRF5, IRF7) and PU.1, rather than by NF-κB as with hBD-2 and hBD-3 [27].

Another function of β-defensin-1 may be to cooperate in the induction of other β-defensins, such as β-defensin 2 and β-defensin 3, which the data in this study supports. To demonstrate that multiple β-defensins can contribute to the early bacterial innate immune response in the airway epithelium, the site where they are most abundant in the lung [28,30], we took advantage of the type III secretion system of *B. bronchiseptica*. This type III secretion system contributes to the virulence of the bacterium by inhibiting the activation of NF-κB in the host cells [24,25]. *B. bronchiseptica*, which is an LPS-containing Gram-negative rod that is 5 µm in length, targets the airway epithelium [26] where β-defensins are most abundant, making this a good infection model to study the effects of β-defensins on colonization of the airway. 

By demonstrating that the combination of inhibition of mBD-3 by the virulence factor, the type III secretion system in RB50 of *B. bronchiseptica* and a genetic deficiency of mBD-1, we observed an increase in bacterial colonization of the airway after 24 h. Specifically, the lack of the type III system results in an increase in mBD-3 mRNA levels, which mirrors the in vitro results we obtained with the bovine homologue in cultured tracheal epithelial cells [12]. 

Other studies support the concept of bacterial virulence factors affecting β-defensin production [14]. In human airway epithelial cells, the capsule of *Klebsiella pneumoniae* protects the bacterium from β-defensin bactericidal action and impedes the expression of mBD-4 and mBD-14, for mutant bacteria lacking the capsule induce higher levels of β-defensin-3, -4 and -14 in vivo than the WT bacteria. In addition, the concept of β-defensins and other antimicrobial peptides working together as therapeutic agents has also been demonstrated [49,50].

The contribution of antimicrobial peptides in *B. bronchiseptica* has not been fully examined in the distal lung and in adaptive immunity but this was not the purpose of our study. Our current study focused solely on the ability of a virulence factor to influence β-defensin expression in TEC and to study their coordination in impeding colonization of the trachea. This type III secretion factor also affected the presence of neutrophils in the TEC and the ability to mount an early specific immunoglobulin immune response. mBD-1 also contributed to the initial immune response, coordinating with mBD-3 to impede colonization and to assist in mBD-3 mRNA expression. Other β-defensins and antimicrobial peptides may also work together to be complimenting the overall innate immune response of the upper airway, preventing the infection from colonizing and progressing to disease in the distal lung.

## 5. Conclusions

The results of this research demonstrate that β-defensins coordinate to prevent colonization of the trachea with a pathogen that targets tracheal epithelial cells, *Bordetella bronchiseptica,* and affect the local recruitment of neutrophils. The results also demonstrate the influence of virulence factors on β-defensin 3 gene expression in vivo by the host and that host factors such as β-defensin 1 can influence the expression of other β-defensins.

## Figures and Tables

**Table 1:** Primers for qRT-PCR.

Gene	Primer Sequences	Product Size
mBD-3	Forward: GCATTTCTCCTGGTGCTGCTGTCTC	128 bp
	Reverse: CTGCCAATCTGACGAGTGTTGCC	
mBD-4	Forward: CTCACTTGCAGCCTTTACC	202 bp
	Reverse: CATGGAGGAGCAAATTCTGG	
mBD-14	Forward: CCTCATCTTGTTCTTGGTGCCTGCTG	102 bp
	Reverse: TTAAGTACAGCACACCGGCCACCTCT	
KC	Forward: ATGGCTGGGATTCACCTC	166 bp
	Reverse: CACCTTTTAGCATCTTTTGGA	
β-actin	Forward: ATCCTGAAAGACCTCTATGC	287 bp
	Reverse: AACGCAGCTCAGTAACAGTC	
mβ2-µglobulin	Forward: CTCCGTGGCCTTAGCTGTG	69 bp
	Reverse: TTTGGAGTACGCTGGATAGCCT	
